# HSP expression depends on its molecular construction and different organs of the chicken: a meta-analysis

**DOI:** 10.1038/s41598-022-18985-0

**Published:** 2022-09-01

**Authors:** Sharif Hasan Siddiqui, Mousumee Khan, Hosung Choe, Darae Kang, Kwanseob Shim

**Affiliations:** 1 Center for Musculoskeletal Research, School of Medicine and Dentistry, University of Rochester Medical Center, Rochester, NY 14642 USA; 2 Department of Biomedical Science and Institute for Medical Science, Jeonbuk National University Medical School, Jeonju, 54907 Republic of Korea; 3 Department of Animal Biotechnology, College of Agriculture and Life Science, Jeonbuk National University, Jeonju, 54896 Republic of Korea; 4 Department of Agricultural Convergence Technology, College of Agriculture and Life Science, Jeonbuk National University, Jeonju, 54896 Republic of Korea

**Keywords:** Animal biotechnology, Expression systems

## Abstract

Heat shock proteins (HSPs) expression protect the cell from stress, this expression varies on tissue and stress level. Here, we investigated the structure and functional expression of HSPs in different chicken organs using meta-analysis. A total of 1253 studies were collected from three different electronic databases from January 1, 2015 to February 1, 2022. Of these studies, 28 were selected based on the specific criteria for this meta-analysis. The results for the expression of HSPs and the comparative expression of *HSPs (HSP90, HSP70, and HSP60*) in different chicken organs (brain, heart, liver, muscle, and intestine) were analyzed using the odds ratio or the random-effects model (REM) at a confidence interval (CI) of 95%. Compared to the thermoneutral groups, heat stress groups exhibited a significant (*P* < 0.01) change in their *HSP70* expression in the chicken liver (8 trials: REM = 1.41, 95% CI: 0.41, 4.82). The expression of different HSPs in various chicken organs varied and the different organs were categorized according to their expression levels. HSP expression differed among the heart, liver, and muscle of chickens. HSPs expression level depends on the structure and molecular weight of the HSPs, as well as the type of tissue.

## Introduction

Heat stress has a negative impact on the health and welfare of animals^1–4^. Owing to global warming, the severity of heat stress has increased^5–7^. Additionally, heat stress affects the physiological functions of livestock, leading to poor performance^8,9^. Specifically, chickens are more sensitive to heat stress than other animals because they lack sweat glands, possess a high metabolic rate, and their bodies are covered with heavy feathers^10,11^. However, in recent years, the demand for chicken has increased to fulfill the global protein requirements^12,13^. However, it is difficult to increase chicken production because of global warming^14^. It has been reported that heat stress in cells is reduced by the high expression of molecular chaperones known as heat shock proteins (HSPs)^15,16^.

HSPs are proteins that are synthesized in response to heat stress to protect cells^17,18^. Under heat stress, heat shock factors (HSFs) are translocated from the cytoplasm to the nucleus, where they bind to heat shock elements (HSEs) to synthesize HSPs which protect the cell by preventing protein aggregation, misfolding, and unfolding^19–21^. The HSP family has been classified as small and large molecular weight HSPs, with molecular weights 8–28 kDa and 40–105 kDa respectively, which are found in different regions of cells^22–24^. HSP90 and HSP70 are well-known chaperones that function well^23^. Furthermore, HSP40 interacts with HSP70 and acts as a co-chaperone^25–27^. It is well known that all HSPs function to prevent cell death. However, their expression levels depend on their structure and localization^23,28^. Additionally, the expression levels of some HSPs remain unclear. Several issues are thus of concern. First, there is a lack of comparative research on different chicken organs. Second, HSPs of the HSP family are more pronounced in certain organs or tissues of the chicken. Third, depending on the expression level of HSPs, the relationship between the different organs of the chicken and heat tolerance is unknown.

In this study, we discuss the function of HSPs based on their structure and localization in cells. We then discuss the set of meta-analyses performed to explore different HSP expressions in five different chicken organs under heat stress. Finally, we list the statistical analyses perfumed to determine the relationship among different organs of chickens for thermo-tolerance based on molecular chaperone expression during heat stress conditions. Several studies have investigated the effects of heat stress on immunoglobulin and HSP expression^29,30^. To the best of our knowledge, this meta-analysis is the first to explore the differential expression of HSPs in different chicken organs and their relationship. Therefore, our study was conducted to determine the expression of HSPs depending on their structure and the different organs in which they are encoded.

## Results

### Study retrieval and selection

The literature searches and selection procedures are illustrated in Fig. S1. A total of 1253 studies were collected from different electronic databases. We excluded 523 studies for repeated selection of the same literature. We shortlisted 159 studies related to our study. Finally, 28 studies were selected based on their suitability with the hypothesis of our meta-analysis. The selected literature in our study is characterized in Table S1. The quality of all the selected articles was evaluated using the PEDro scale (Fig. S2). The selected articles were considered of moderate quality for this meta-analysis.

### Protein–protein interactions network of HSPs

**Figure 1:**
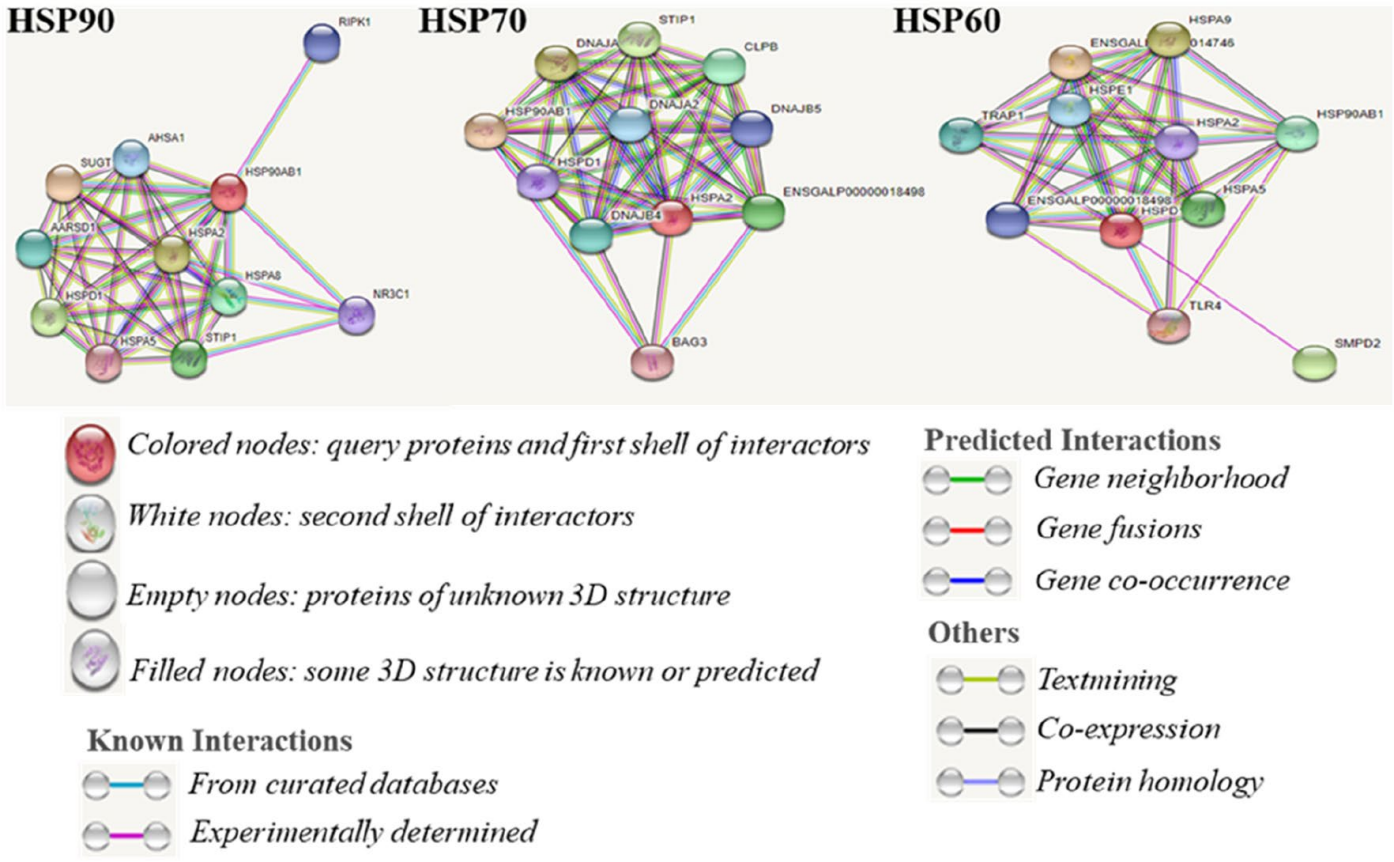
Protein–protein networks constructed by the STRING analysis.

In a cell, when different entities of a protein bind to each other, the protein becomes functional and plays an important role in the cellular processes (Fig. 1). We used the STRING database to identify and analyze the HSPs as well as to define how HSPs function in different ways^31^. HSP90 affects several entities, among which receptor-interacting protein kinase 1 (RIPK1) plays a crucial role in enhancing cell viability by suppressing apoptosis and necroptosis^32^. The activator of 90 kDa HSP ATPase homolog 1 (AHSA1) is an important chaperone of HSP90, which regulates osteosarcoma invasion^33^. In HSP70, Bcl2-associated athanogene 3 (BAG3) promotes cell adaptability under stressful conditions by stabilizing cytoskeletal integrity^34^. In *HSP70*, Bcl2-associated athanogene 3 (BAG3) promote cell adaptability during stressful condition by making cytoskeletal stability^35^. CLPB promotes heat stress resistance activity under heat stress^36^. HSP A2 (HSPA2) is a testis-specific protein that increases fertility^37^ . TLR4 protein lessens the inflammatory reaction during stress conditions, while HSP60 induces TLR4^38^. SMPD2 reduces stress severity in the endoplasmic reticulum by regulating the cell cycle^39^. TRAP-1 regulates oxidative phosphorylation by inhibiting succinate dehydrogenase^40^.

### Transmembrane region and disorder sequence regulates cycle phase of HSPs expression

**Figure 2:**
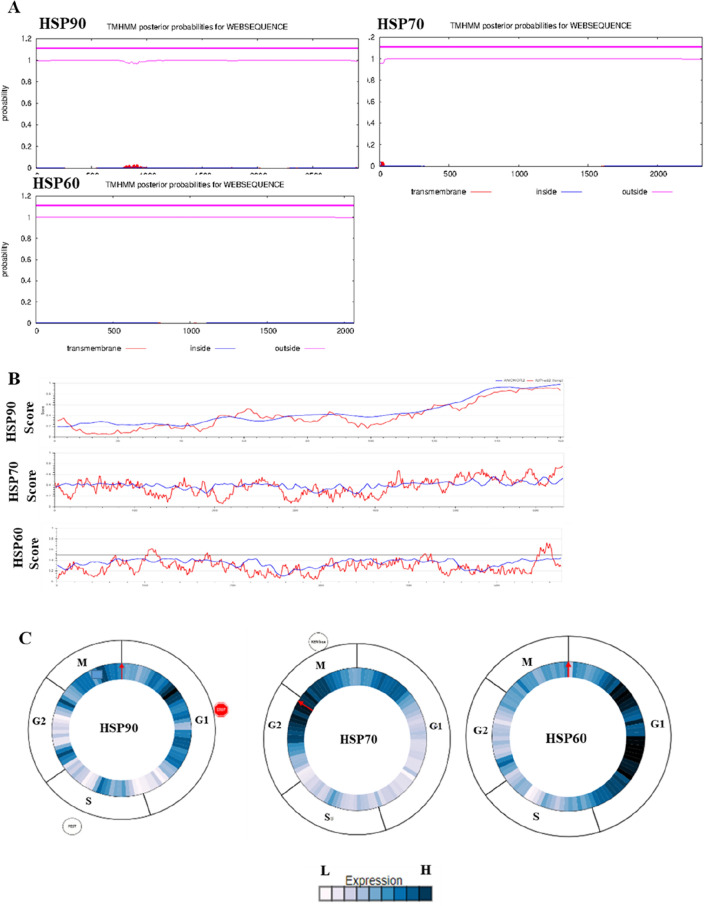
Prediction of transmembrane regions of HSPs. (**A**) Transmembrane regions are shown in red, and other regions are predicted to be either outside (pink) or inside (blue) the membrane. (**B**) Disorder profiles of HSPs sequences. (**C**) HSPs protein expression levels in a four-stage model of the cell cycle.

The function of a protein depends on its region of origin. This function leads to the translocation of polypeptides into different organelles in the cell. Therefore, transmembrane location is important for protein function. However, we did not find any transmembrane regions in *HSP90, HSP70*, or *HSP60* (Fig. 2A). DNA replication slippage and recombination are responsible for unstable low-complexity regions. However, this does not mean that all the disordered regions contain low-complexity sequences, and secondary structures are also responsible for this region (Fig. 2B). The sequence transformation of HSPs depends on the ligands of the secondary structure of the HSPs. Our results showed that *HSP70* and *HSP60* possessed higher expression levels than *HSP90*. HSP90 was expressed in the G1 phase of the cell cycle; HSP70 was expressed during the M and G2 phases; and HSP60 was highly expressed during the G1 phase of the cell cycle (Fig. 2C).

### Analysis of studies that investigate HSPs mRNA expression level and study biases

**Figure 3:**
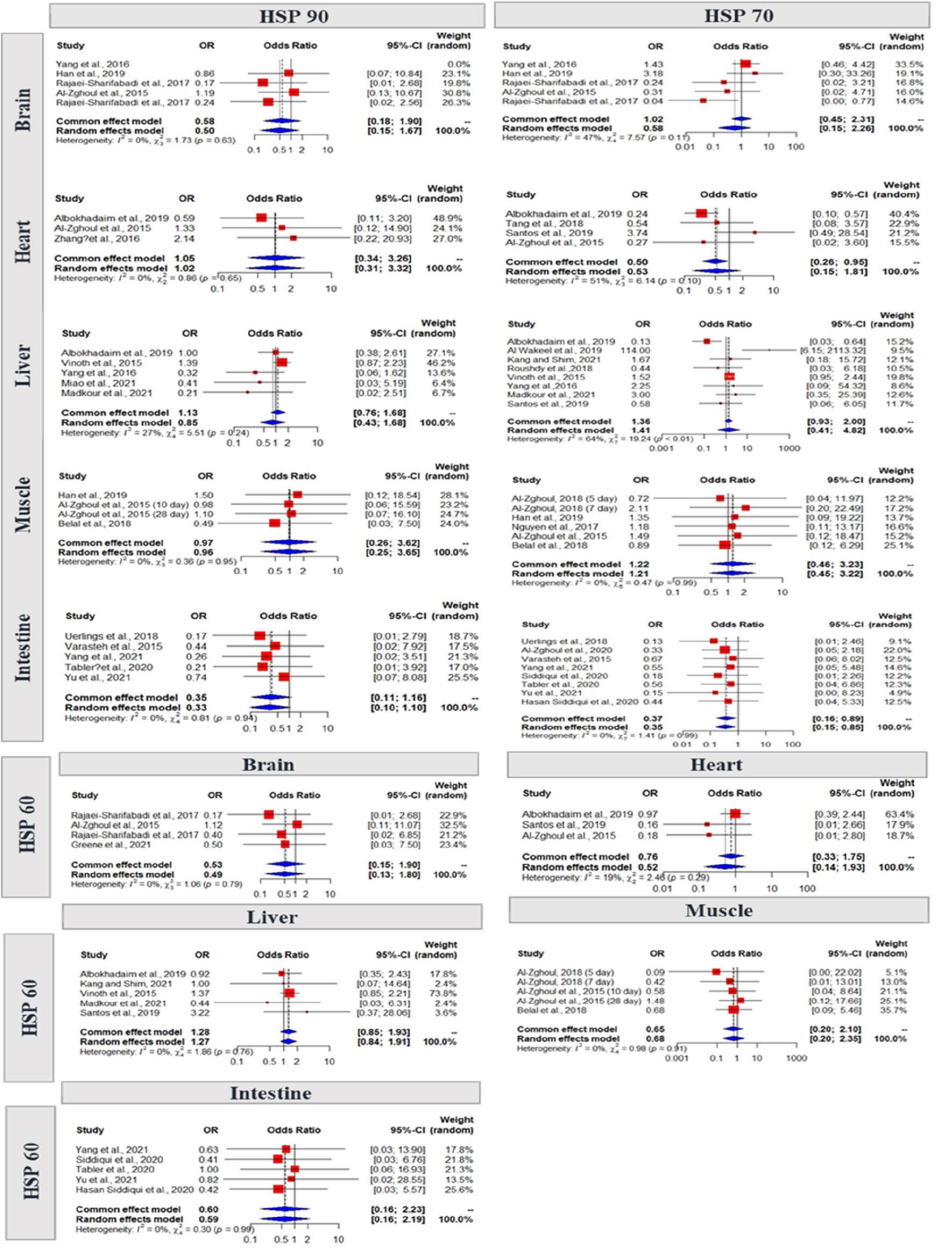
Forest plots representing the effect of heat stress on HSP90, HSP70 and HSP60 gene expression in different organs of chicken.

**Figure 4:**
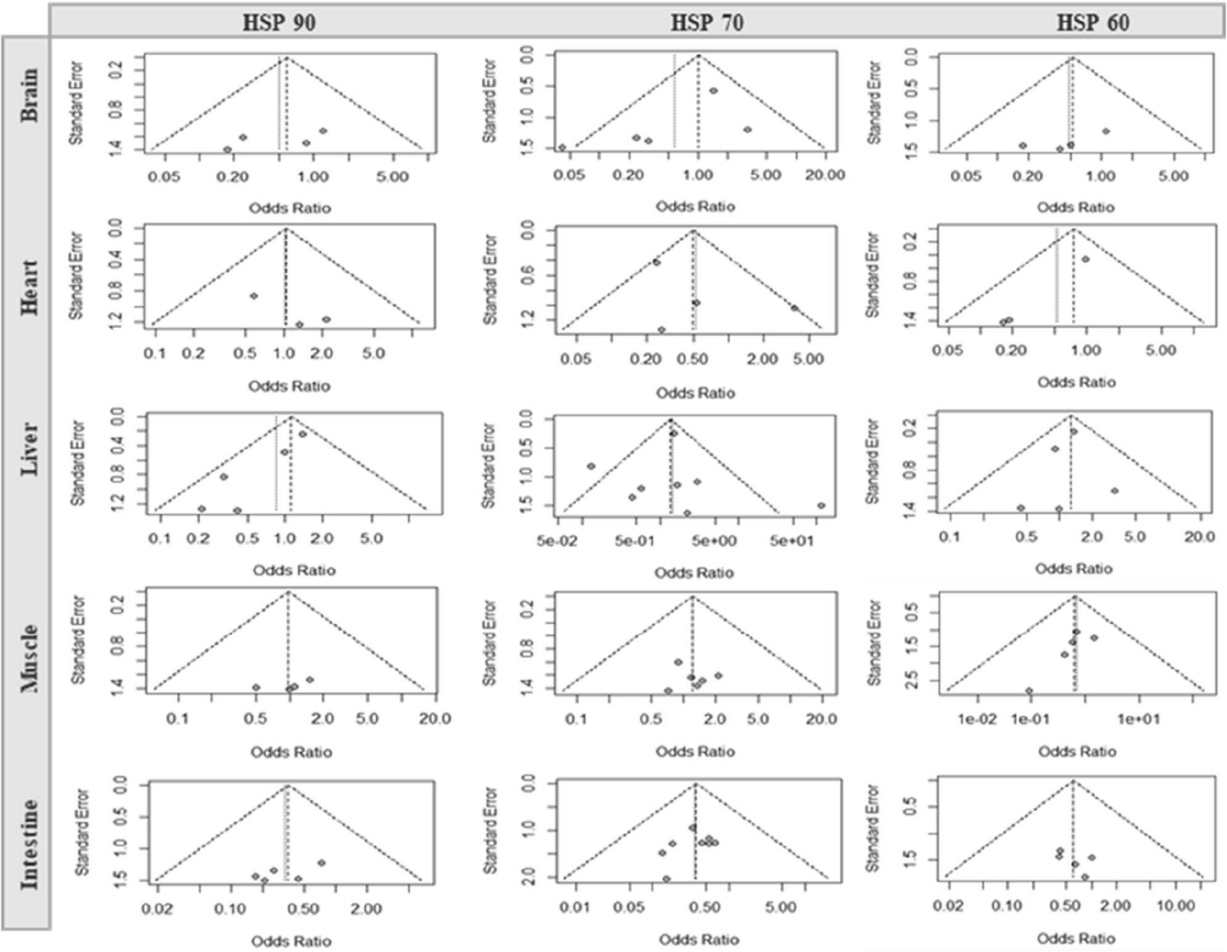
Funnel plots representing the effect of heat stress on different organs mass journal publication bias.

A total of 28 studies reported comparative HSP expressions in different chicken organs under thermoneutral conditions and heat stress. Significant heterogeneity was found in different HSPs in different organs, indicating that the random effect model was I2 = 27%, OR = 0.96, 95% Cl = 0.25, 3.65 of HSP90 in the liver; I2 = 47%, 51%, 64%; OR = 0.58, 0.53, 1.41; 95% Cl = 0.15, 2.26; 0.15, 1.81; 0.41, 4.82 of HSP70 in the brain, heart, and liver, respectively; I2 = 19%; OR = 0.52; 95% Cl = 0.14, 1.93 of HSP60 in the heart. Additionally, the gene expression of HSP70 in the liver was significantly (*P* < 0.01) different between the thermoneutral and heat stress groups in our study (Fig. 3). We assessed the publication bias of the selected articles using funnel plots. The funnel plot of this study indicates that all the studies were asymmetrical, which means that there was no publication bias except for HSP70 in the liver study publication (Fig. 4).

### Relative expression of HSPs mRNA during heat stress

**Figure 5:**
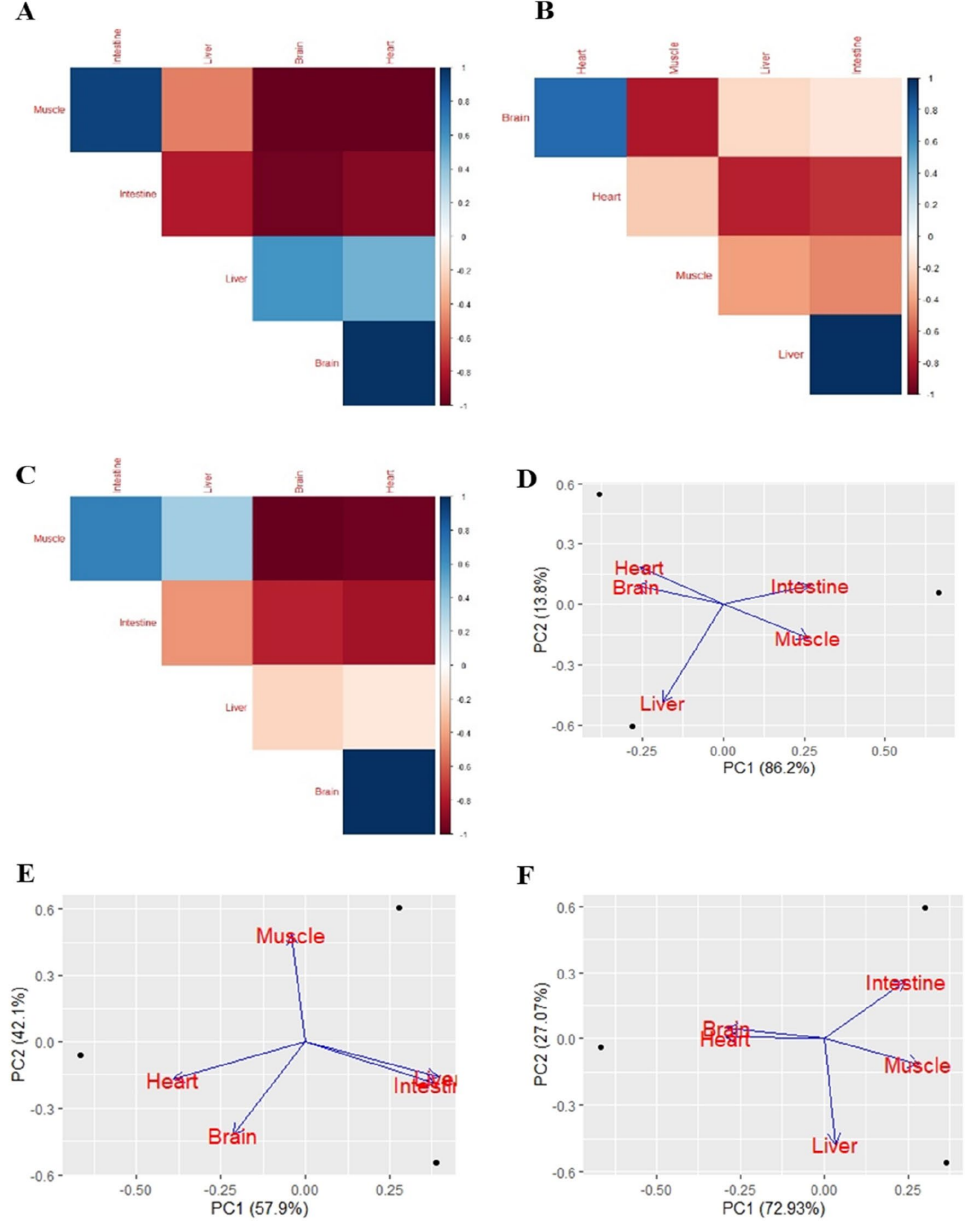
Effect of heat stress on heat shock protein expression in different organs of chicken. (**A–C**) Correlation among different organs of chicken in HSP90, HSP70, and HSP60 respectively. (**D–F**) Principal component analysis (PCA) among different organs of chicken in HSP90, HSP70, and HSP60 respectively.

The mRNA expression of HSPs in different organs of chickens was collected from the selected literature. Heat stress changed the mRNA expression compared to the thermoneutral group. We used a coefficient correlation analysis to determine the relationship between the two different organs of the chicken and analyzed the correlation of five different organs with three different HSPs (*HSP90, HSP70*, and *HSP60*). For *HSP90*, muscles with the intestine, as well as heart with the brain, were positively correlated with each other. In contrast, the brain and heart were negatively correlated with muscle, and the intestine was negatively correlated with the brain (Fig. 5A). For *HSP70*, the liver, intestine, and heart were positively correlated with the brain. In contrast, the liver and intestine were negatively correlated with the heart, while the brain was negatively correlated with the muscle (Fig. 5B). For *HSP60*, the heart and brain were positively correlated. Nonetheless, the brain and heart were negatively correlated with muscle mass (Fig. 5C).

We performed principal component analysis (PCA) on the expression of HSPs to investigate the relationship between the five different organs of the chicken. For *HSP90*, the first two principal components (PCs) showed a total variance of 100% among the five organs (PC1 = 86.2% and PC2 = 13.8%) (Fig. 5D). The heart, brain, intestine, and muscle were all positively correlated. For *HSP70*, the first two PCs showed a total variance of 100% among the five organs (PC1 = 57.9% and PC2 = 42.1%). The heart and brain were positively correlated, while the intestine and liver were strongly positively correlated (Fig. 5E). For *HSP60*, the first two PCs showed a total variance of 100% among the five organs (PC1 = 72.93% and PC2 = 27.07%) (Fig. 5F).

### Categorized the different organs by HSPs mRNA expression

**Figure 6:**
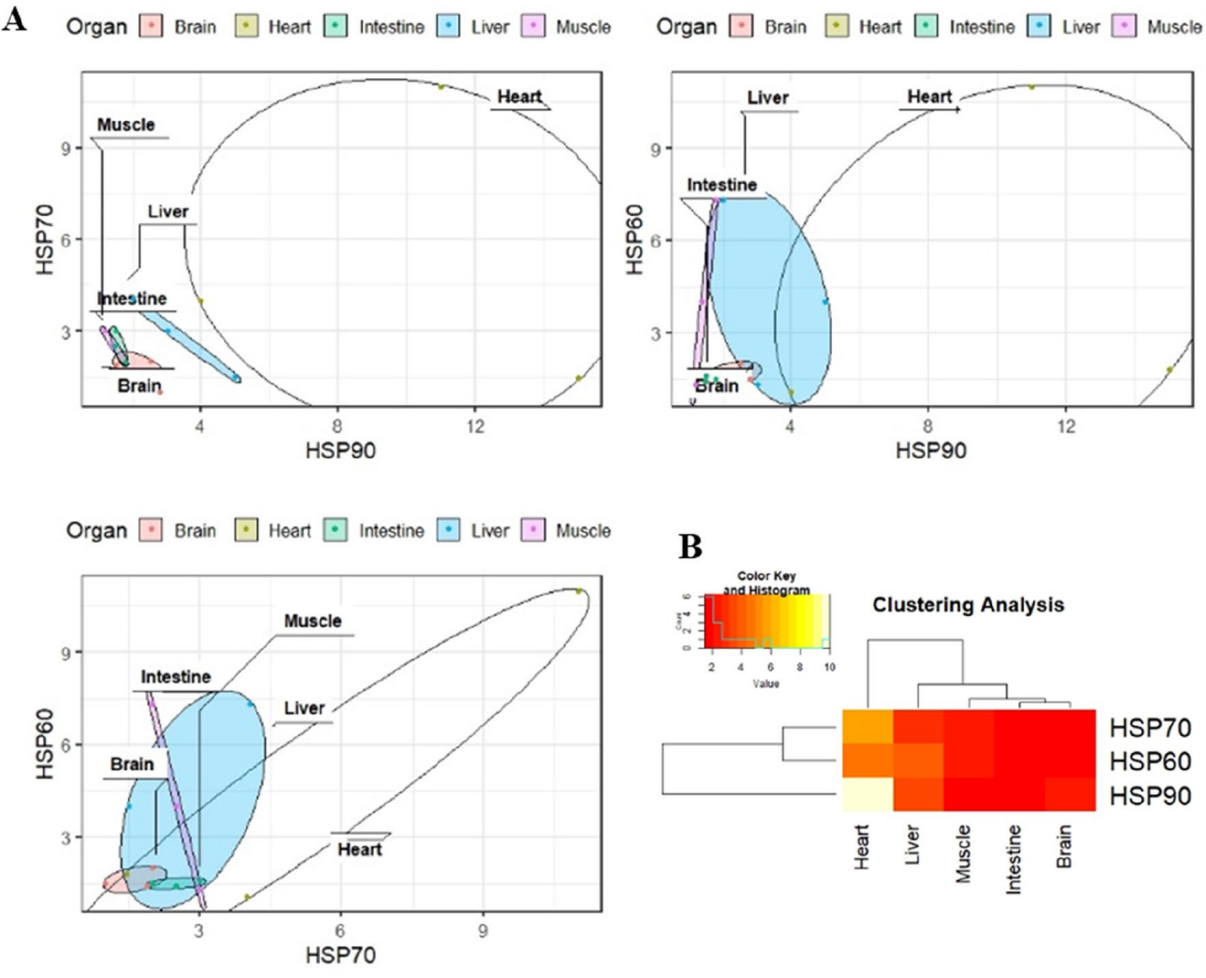
Heat shock proteins expression level and clustering in different organs. (**A**) Different gene expression level in different organs of chicken. (**B**) Clustering heat map indicate the relation between heat shock proteins and different organs.

We categorized the different organs of chickens based on the expression of HSP90, HSP70, and HSP60 and their ratios. Circular plots and cluster heat maps were analyzed for different organs. For the *HSP90* and *HSP70* ratio, the expression ratios were similar in the muscle, intestine, and brain. In contrast, the expression ratio was different in the liver and heart compared to that in the other organs of the chicken (Fig. 6A).

For the *HSP90* and *HSP60* ratio, the expression ratio was similar to those of HSP90 and HSP70, respectively. For *HSP60* and *HSP70* ratio, the expression ratio was similar in the muscle and brain. In contrast, the expression ratio was different in the intestine, liver, and heart compared to that in the other organs of the chicken. Furthermore, we categorized the organs according to their relationship to the mRNA expression of HSPs. HSP expression level in the heart was different from that in the other organs (Fig. 6B).

## Discussion

Several articles have been published in different peer review journals regarding HSP expression in vitro and in vivo^41–45^. However, to the best of our knowledge, this is the first meta-analysis to investigate the different HSP expression levels in different chicken organs. This meta-analysis was performed using 28 published studies in peer-reviewed journals. We observed that the expression levels of the same HSPs differed between organs. Moreover, the expression levels of different HSPs differed in the same organ. This meta-analysis detected low to moderate heterogeneity response factors, and a random-effects model was used for the literature selection procedure with some variation for this study^46^.

The expression levels of HSPs increase under heat stress^47,48^, which assist in protein folding and protein folding repair^49–51^. The functions of different HSPs depend on their molecular weights and structures^52,53^. Our results also showed that each HSP has a unique structure and different function levels. We believe that this structure is responsible for the differential expression and function levels. Moreover, specific HSPs are localized in different cell organelles^24,54^. For instance, *HSP90* localizes to the cytoplasm, *HSP70* to the nucleus, and *HSP60* to the mitochondria (Fig. S3). Their function also different, similar to their individual structure. The function of HSP90 is to assist myosin folding and sarcomere formation, HSP70 promotes protein folding and folding repair, and HSP60 helps form a complex of hetero-oligomeric proteins^24^. Previous study reported that different cell signaling pathways regulate HSPs expression which maintains cell homeostasis during stress conditions^55^.

The duration and intensity of heat stress regulate the expression levels of the HSPs^56^. Moreover, the expression levels of the HSPs are different in different organs^57^. It has been reported that energy metabolism is an important factor in the expression of HSPs^58^. Energy metabolism differs in various organs of animals, depending on energy expenditure. Heat stress regulates energy synthesis by altering glycolysis^59,60^. Therefore, the expression of HSPs is different in various organs under heat stress because of the different heat sensitivities in different organs. A previous study reported that *HSP70* protects against apoptosis under stress conditions^60–62^. Even the HSP70 carries the information of stress to the immune system and induces immunity^63^. HSP60 causes inflammation in the cell, which is induced by stress conditions, thus protecting cells^64,65^. Moreover, *HSP90* expression regulates the cell cycle and cell survival by maintaining different signaling pathways^66,67^. These results indicate that *HSP90, HSP70*, and *HSP60* play important roles in protecting cells and tissues from heat stress. Our study categorized the different organs of chickens based on the expression levels of HSPs. We believe that the organs that are more heat-sensitive express more HSPs. Additionally, different HSPs function in the cell in different ways, owing to their unique structure.

The major strength of this meta-analysis is that we explored *HSP90, HSP70*, and *HSP60* in five different organs of chickens, which systematically validated the effects of heat stress on the expression of HSPs to investigate the protection level of different heat-stressed organs. It has been reported that HSPs expression and function depend on particular tissue and organs as well as develop thermotolerance in those tissues^68,69^. The aim of this study was to categorize heat-sensitive organs and HSP functions to protect chickens from heat stress. The limitation of this study was the small number of studies selected and the inability to differentiate the heat stress temperatures between the same age and sex. Moreover, some studies reported publication bias, which might be a methodological issue.

Although the HSPs belong to the same HSP family, their expression levels are different during heat stress conditions owing to their different structures. Moreover, the expression levels differ in different organs and at different stress levels. Further studies are needed to understand why the expression levels are different in different chicken organs by analyzing the genetic construction of these organs.

## Methods

This meta-analysis was conducted according to the Preferred Reporting Items for Systematic Reviews and Meta-Analyses (PRISMA) criteria^70^. The participants were divided into two groups based on: (1) the structure and function of HSPs and (2) the expression of HSPs in different organs during heat stress.

### Search strategy

We searched previously published papers in three familiar electronic databases. These included the PubMed (National Library of Medicine, Bethesda, Maryland, USA) and Web of Science (Thomson Reuters, London, UK) from January 1, 2015 to February 1, 2022. The enrolled studies must have been published as a research article in a peer-reviewed journal in English, and these studies must have looked at different chicken species. The following keywords were used for the literature search: heat stress, chicken, HSP90, HSP70, HSP60, brain, heart, liver, muscle, and intestine. The amino acid and protein sequences of chicken were searched using the NCBI database (https://www.ncbi.nlm.nih.gov/). The titles and abstracts of the selected literature were recognized by exploring keywords according to the selection conditions.

### Selection criteria

The selected studies were assessed based on their appropriateness and significance. The selection method is illustrated in a flowchart (Fig. S1). Literature was selected for this meta-analysis only when the following requirements were satisfied:

1. The selected literature must have a thermoneutral group for comparison with the heat stress group.
2. The literature must have two parts: heat stress initiation and an endpoint for the assessment of heat stress. Heat stress usually alters the expression of HSPs in different organs of chickens compared with that in the control group.
3. All data in both groups were presented as means in either tables or figures with standard deviation (SD) and/or standard error (SE).
4. All the selected studies were self-paced, which means that the published literature was compiled by the author and discussed in their own way.
5. The effect of heat stress on chickens has been reported in the literature. For instance, when chickens experience heat stress, they exhibit changes in the gene expression of HSPs.
6. The selected literature must be published in an international, peer-reviewed journal. This indicates that we did not consider preprint or unpublished articles for selection. Moreover, every selected article was written in English.

### Study classification

A total of 28 eligible studies were selected from 730 primary peer-reviewed studies that fulfilled the selected conditions. The literature was considered based on vivid environmental or related factors, which were assessed by researchers, such as, the heat stress condition during an animal trial. Two different groups were considered: (i) thermoneutral group and (ii) heat stress group. Moreover, if duplicate studies were detected, we considered the most recent and detailed information-related literature.

### Information extraction

We extracted information from the selected literature individually using predesigned combined consistent reporting forms allocated to the different study areas. These data could not be collected directly and we extracted the type of data using the Plot Digitizer software (http://plotdigitizer.sourceforge.net/). For the appropriate meta-analysis, the following information was collected: data source (first author, publication year), chicken characteristics (species, age, strain, and number), study design, sample size, data extraction (*HSP90, HSP70, HSP60*), and description of the data. We considered different organs for our selection, including the brain, heart, liver, muscles, and intestine. For risk assessment, we analyzed the accuracy of the variables. Nonetheless, data accuracy was affected by the variation in the HSP gene expression in different organs of the chicken and by accustomed covariates. For the meta-analysis, the level of agreement of both variables and analysis of the correlation coefficient were understood using the Kappa coefficient^71^. For instance, we used a cast-off Kappa coefficient to analyze the inter-rater reliability between different groups. This analysis helped evaluate the same phenomenon between the thermoneutral and heat stress groups. The analysis revealed a close correlation between the thermoneutral and heat stress groups (interaction correlation coefficient = 0.99; 95% confidence interval [CI]: 0.99 and 0.99). Therefore, we were assured that there were minimal study biases measured during the extraction of information for the meta-analysis^72^.

### Study quality assessment

The selected literature was assessed following our previous study^29^ using the Physiotherapy Evidence Database (PEDro) scale, while the inclusion criteria of this study were not related to this scale. Nonetheless, the PEDro scale assists in analyzing the statistical information of primarily selected journals and helps in selecting effective literature for meta-analysis^73^. This scale analyzes the quality of the literature based on 11 criteria associated with the experimental design. The scale ranged from 0 to 10. A high-quality article was given a score ≥ 7, a moderate quality article was scored 5–6, and a poor quality article was scored ≤ 4^74^.

### Statistical analysis

Statistical analysis was conducted using the “metafor” package^75^ in R V 4.1.0 (Vienna, Austria: R Foundation for Statistical Computing). Our aim was to compare the expression of HSPs between the thermoneutral and heat stress groups in different chicken organs. The effect size of the incessant results was evaluated using a random-effects model (REM) with a 95% CI, and an odds ratio (OR) was used to obtain dichotomous results. Heterogeneity among the selected studies was evaluated using Cochran’s Q statistic, and the I2 test and bias of selected articles were analyzed using funnel plots. We considered heterogeneity when P = 0.05 (Q statistic) and I2 was 50. In contrast, the random- and fixed-effects models were considered for the analysis. Publication bias of the selected articles was analyzed using Begg’s test following the Cochrane Handbook^76^. Publication bias was considered at P < 0.05. Furthermore, we used the “ggplot2” packages of the R software for comparative analysis of HSP expressions in different organs of the chicken.

## Supplementary Information

Supplementary Information 1.

Supplementary Information 2.

Supplementary Information 3.

## Data Availability

All data generated or analyzed during this study are included in this published article [and its supplementary information files].
