# Supplementary material for: HSP expression depends on its molecular construction and different organs of the chicken: a meta-analysis
Source: Sci Rep. 2022 Sep 1;12:14901. doi: 10.1038/s41598-022-18985-0 (PMC9437049; doi:10.1038/s41598-022-18985-0)
Supplement: Supplementary file 1 — Supplementary Information 1. [file 41598_2022_18985_MOESM1_ESM.docx]

Gene Sequencing

>NM_001109785.2 Gallus gallus heat shock protein 90 alpha family class A member 1 (HSP90AA1), mRNA

GGGGCGCGCACTAGTGGAGGCGCAGCGTTGAGGGTGTAGCGCGTTTGCTGATTCTGTGAGGAGACCGACA

GCCCTTCCCCCGCTGCCAAGATGCCAGAAGCTGTGCAAACACAGGACCAACCAATGGAGGAGGAAGTGGA

GACCTTTGCCTTCCAGGCTGAGATTGCTCAGTTGATGTCTCTGATTATCAACACTTTTTACTCCAATAAG

GAAATCTTCTTGAGGGAACTGATCTCCAATTCATCTGATGCTCTGGACAAGATCAGATATGAGAGTTTGA

CTGACCCGAGCAAGCTGGATTCTGGAAAAGACCTGAAAATTAACCTGATTCCAAACAAGCATGATCGCAC

TCTGACCATTGTGGATACCGGCATAGGGATGACCAAAGCTGACCTTGTCAACAATCTTGGTACTATTGCC

AAGTCTGGTACCAAGGCTTTCATGGAAGCACTGCAGGCAGGGGCTGATATTTCCATGATTGGTCAGTTTG

GTGTTGGTTTCTACTCTGCTTACCTTGTTGCGGAGAAGGTGACAGTGATCACCAAGCACAATGATGATGA

GCAGTATGCTTGGGAGTCATCAGCTGGAGGATCTTTCACTGTCAGACTTGATAACGGTGAACCTTTGGGC

CGTGGAACAAAAGTTATCCTGCACCTCAAAGAAGACCAAACTGAATATCTGGAAGAACGGCGAATCAAGG

AGATTGTGAAGAAGCATTCTCAGTTCATTGGCTACCCTATTAGGCTCTTTGTGGAGAAGGAGCGCGATAA

GGAGGTGAGTGATGATGAAGCTGAGGAAAAGGAGGAGGAAAAAGAGGAGAAGGAGGAGAAGACAGAAGAT

AAACCAGAGATTGAGGACGTTGGTTCTGATGAGGAAGAGGAAAAGAAGGATGGAGATAAGAAGAAGAAAA

AGAAGATCAAGGAGAAGTACATTGATGAGGAAGAGCTCAACAAGACCAAGCCTATTTGGACCAGGAACCC

AGATGACATCACCAATGAGGAGTACGGGGAGTTCTATAAGAGCCTAACTAATGACTGGGAGGACCACTTG

GCTGTCAAACACTTCTCTGTGGAAGGTCAGCTGGAATTCAGAGCTCTCCTGTTTGTCCCACGACGTGCAC

CTTTTGATCTGTTTGAAAACAGGAAGAAGAAAAACAACATCAAGCTCTATGTACGCAGAGTTTTCATCAT

GGACAACTGTGAGGAACTGATCCCCGAATACCTGAACTTCATGAGAGGTGTCGTAGACTCTGAGGATTTA

CCTCTGAATATTTCTCGTGAAATGCTGCAACAAAGCAAGATCCTTAAAGTGATTCGGAAGAACTTGGTGA

AGAAGTGTTTGGAACTTTTCACTGAGTTGGCTGAAGACAAGGAGAACTACAAAAAGTTCTATGAGCAGTT

CTCCAAGAACATCAAGCTTGGAATACATGAAGACTCCCAGAACCGCAAGAAACTCTCAGAGTTACTCAGG

TATTACACATCTGCATCTGGTGATGAAATGGTTTCTCTGAAGGACTACTGCACTCGCATGAAGGAAAACC

AGAAACATGTCTACTACATCACTGGTGAGACAAAGGACCAGGTGGCTAACTCTGCTTTTGTGGAGCGCCT

TCGCAAGCATGGCCTGGAAGTGATCTACATGATTGAGCCTATTGATGAATATTGTGTGCAGCAGCTGAAG

GAATTTGAAGGCAAGACTCTGGTTTCTGTAACAAAAGAGGGCTTAGAGCTTCCAGAAGATGAAGAAGAGA

AGAAGAAACAGGAGGAGAAAAAAGCCAAGTTTGAAAACCTTTGCAAAATTATGAAAGATATCCTTGAGAA

GAAAGTAGAAAAGGTTGTTGTGTCCAATCGCTTGGTAACTTCTCCATGCTGTATTGTAACAAGTACATAT

GGCTGGACTGCCAATATGGAGAGGATTATGAAGGCACAGGCTTTGAGAGACAACTCCACAATGGGATACA

TGGCAGCAAAGAAGCACCTGGAGATCAATCCTGATCATTCCATCATTGAAACACTGAGGCAGAAGGCAGA

GGCTGATAAGAATGACAAATCTGTGAAGGATCTTGTCATACTGCTGTACGAGACAGCTCTCCTGTCCTCT

GGCTTTAGTTTAGAAGATCCCCAGACACATGCCAACCGCATTTACAGAATGATCAAACTTGGCCTGGGCA

TTGATGAAGATGATACTGCTGCTGAGGAGGCCAGTCCTGCAGTTACCGAGGAGATGCCACCTCTGGAAGG

TGATGATGACACATCACGCATGGAGGAGGTGGATTAAAACAGTTTACAGGAACTCATGAATGTTTCCTTG

GCTAATATGAATAAGTTATATTTTGTATATTATGAATGTTACCTGCCAAAAAAAAACAAAAATCTTTGAC

ACTTTTGTCTGCATTCCCTCTTTATATTTATTTTCAAAGATGTTATACCTTTATTTTTGTTACATTGCTT

TTTCAGCTGATGTGAGATACAAATGCCATTGAGGGAGTATTTTCTTTAACACTGTACAACCCTAGACAGG

CAAGTAAGGAGTAGTTATTTTTGTCTGTATTAGCTGGTTTGCAGGTGGCAGGGTTTTTGACTTATTCTTA

ATTGCCAGAAAAGTAACAAAGACGGTATGACATCTGGGTGTTCGAGTTTGTTTAGTGTTATACAGCTCTT

CAACTCTGAATATGTCTAACATAGAGTACCTAGTAACTAGGTATCTATGAGAGCTGACACTTGGGAAGCT

TTAACCCTTTTGCTCCTAACAAGGTCTTGATGTTTAAAGTTGTTTTTTATACTGTTCAAGGATTCTGGAT

TGCACTCTACCATAGAATAGAATCCACTGTAAATCTCTATTGTGACTTATGCAAATCTGCATGTACAGTT

CAAACCTAGAACTGTAAGAATAAAAGTGTTAAGAATGAGTTGTTGC

>NM_001006685.1 Gallus gallus heat shock protein family A (Hsp70) member 2 (HSPA2), mRNA

ACGGCAGATCGCGCCGCAGACAGCAGCGAGAAGCGGGCGGAGGAGACGTGACTGCGAGCGAGCAAGTGAC

TGGCGGAGCGAGTGGCTGACTGACCAAGAGGAATCTATCATCATGTCTGGCAAAGGGCCGGCCATCGGCA

TCGATCTGGGCACCACGTATTCTTGCGTGGGTGTCTTCCAGCATGGCAAAGTGGAGATCATTGCCAACGA

CCAGGGGAACCGCACCACACCCAGCTATGTGGCCTTCACCGATACAGAGCGCCTCATCGGGGATGCTGCC

AAGAACCAAGTGGCAATGAACCCCACCAACACCATCTTTGATGCCAAGCGTCTCATCGGCCGCAAGTATG

ATGACCCCACAGTGCAGTCAGACATGAAGCACTGGCCCTTCCGTGTGGTGAACGAGGGTGGCAAGCCCAA

GGTGCAGGTGGAGTACAAGGGTGAGATGAAGACCTTCTTCCCAGAGGAGATCAGCTCTATGGTGCTCACC

AAGATGAAGGAGATTGCTGAGGCCTATCTGGGAAAAAAGGTAGAGACTGCTGTTATCACAGTGCCCGCTT

ACTTCAACGACTCCCAGCGCCAGGCCACCAAAGATGCTGGCACCATCACTGGGCTTAACGTGATGCGTAT

TATCAATGAGCCCACAGCAGCTGCTATTGCCTATGGCTTGGATAAGAAAGGTACCCGGGCTGGAGAGAAG

AATGTGCTCATCTTTGACTTGGGAGGGGGCACTTTTGATGTGTCCATCCTTACCATTGAGGATGGCATCT

TTGAGGTGAAGTCCACAGCTGGGGACACCCACCTAGGTGGGGAGGACTTTGACAACCGCATGGTAAACCG

TTTTGTAGAAGAGTTCAAGGGTAAGCACAAGCGTGACAATGCTGGCAATAAGCGAGCAGTGAGGCGTCTG

CGTACAGCTTGTGAGAGGGCGAGGCGTACTCTGAGCTCTTCCACGCAAGCCAGCATTGAGATTGACTCCC

TCTTTGAGGGCATTGACTTCTACACCTCCATCACTCGTGCCCGCTTTGAGGAACTCAATGCTGATCTTTT

CCGTGGTACCCTGGAGCCAGTGGAGAAGGCCCTGCGTGATGCCAAGCTTGATAAGGGCCAGATCCAGGAG

ATTGTGCTTGTGGGGGGCTCCACTCGTATTCCTAAGATCCAGAAGTTGCTGCAAGATTTCTTCAATGGCA

AAGAGCTGAACAAGAGCATCAATCCAGATGAAGCTGTTGCTTATGGTGCCGCTGTGCAAGCAGCTATCCT

CATGGGAGACAAGTCTGAAAATGTGCAAGATCTGCTCCTGTTGGATGTCACCCCCCTGTCCCTGGGCATC

GAGACAGCTGGTGGAGTGATGACTGCTCTCATCAAGCGTAACACCACCATTCCCACCAAACAAACACAGA

CCTTCACCACCTACTCAGACAACCAGAGCAGTGTCCTCGTCCAGGTGTATGAAGGTGAGAGGGCTATGAC

AAAGGACAACAACTTGCTGGGCAAGTTTGACCTAACAGGCATCCCCCCGGCACCCCGTGGAGTTCCTCAG

ATCGAGGTCACTTTTGACATAGATGCTAATGGTATCCTGAACGTCAGTGCTGTGGACAAGAGTACAGGGA

AGGAGAACAAGATAACCATCACCAATGACAAGGGTCGCCTTAGCAAAGATGATATTGACCGTATGGTACA

AGAAGCAGAGAAATACAAAGCAGAGGATGAAGCCAACAGAGATAGGGTGGGAGCCAAGAACTCCCTTGAG

TCGTATACTTACAACATGAAGCAGACAGTGGAGGATGAGAAACTGAAGGGAAAGATCAGTGACCAGGACA

AGCAGAAAGTGCTCGACAAGTGCCAGGAGGTGATCAGTTCGCTTGACCGAAACCAGATGGCAGAGAAAGA

AGAGTATGAGCACAAGCAGAAAGAGCTGGAGAAACTCTGCAACCCGATTGTCACAAAACTGTACCAGGGA

GCTGGAGGAGCTGGGGCAGGTGGCTCCGGTGGCCCAACCATTGAAGAAGTAGATTAAAAAGACTCTTAAA

CTATAGACTGGTTTATGGACAGTCACTCCATTCTTTGCTTTATATTTTTTTCTAACGTTTAAGGAAAAAC

GTCATTGCCAATAACAGAGTTTATTCTGTTGGGTGTGTATAAAGGCAAATCTATCAGCTTGTGGTTTTGA

TAAAAGGGAAGGCACGTCCTGCTTTATAAGGTTAGTAATAGACAAGTTTTGTTAATTCAGATACAGCTCC

TTGTATTCTGGATGTTTGTCTCTGTTTAAATGTCTCTTCTAAAGTAACCACTCGACTGTTGCAGTTGACA

AGTTTCAAGTTAT

>NM_001012916.3 Gallus gallus heat shock protein family D (Hsp60) member 1 (HSPD1), mRNA; nuclear gene for mitochondrial product

GAGTGAGTGCCGCGCGCTTCCCGCCCCTGCGTGCAGATCTGCGTGCCGCGCGCTCGCATCGGACCGGACG

GCGGAGGCGCCTCCCGTAACCCCGCAGACATGCTCCGTTTGCCTGCAGTACTCCGCCAGATCAGGCCGGT

GTCCAGAGCGCTCGCCCCGCACCTCACGCGGGCGTACGCGAAGGATGTGAAGTTCGGTGCGGACGCCCGA

GCCCTGATGCTGCAGGGAGTGGATCTGCTGGCAGATGCCGTAGCTGTCACCATGGGGCCCAAGGGAAGAA

CAGTTATTATTGAACAAAGCTGGGGGAGTCCCAAAGTGACAAAAGATGGCGTGACAGTGGCAAAGGCAAT

CGACTTAAAAGACAAATACAAAAATATTGGAGCCAAATTAGTTCAAGATGTTGCCAATAATACAAATGAA

GAGGCAGGAGATGGTACTACTACTGCAACGGTACTTGCACGTGCGATTGCCAAAGAAGGCTTTGAGAAGA

TCAGCAAAGGAGCTAATCCAGTGGAAATCAGGAGGGGGGTGATGCTTGCAGTTGATGCTATTACAGCTGA

ACTGAAGAAGCTGTCTAAACCAGTTACAACTCCAGAAGAAATTGCACAGGTTGCCACAATATCAGCGAAT

GGAGATCAGGAAATTGGCAATATAATTTCTGATGCCATGAAAAAGGTTGGACGGAAAGGTGTAATCACTG

TCAAGGATGGAAAAACACTAAATGATGAATTAGAAATCATTGAAGGTATGAAGTTTGACCGAGGCTACAT

CTCTCCCTATTTTATTAATACAGCCAAAGGGCAGAAATGTGAATTCCAGGATGCTTACGTCTTAATCAGT

GAAAAGAAGATTTCCAGTGTACAGTCCATAGTTCCAGCTCTTGAAATTGCCAATAGTCACCGCAAACCTT

TGGTCATTATTGCTGAAGATGTTGATGGAGAGGCCCTCAGCACTCTAGTCTTGAACAGACTCAAGGTTGG

TCTTCAGGTTGTTGCTGTAAAAGCACCAGGTTTTGGTGACAACAGGAAAAACCAGCTTAAGGATATGGCA

ATTGCTACAGGTGGTGCTGTGTTTGGAGAGGAGGGTTTGAGCCTAAATGTAGAAGATATTCAGCCTCATG

ATTTTGGAAAAGTTGGAGAGGTCATTGTGACCAAAGATGACACCATGCTTCTAAAGGGGAAGGGTGAGAA

GGCTCAGATTGAAAAGCGCATTCAAGAAATTATTGAACAGCTAGAAGTTACCACAAGTGAATATGAAAAA

GAGAAACTGAATGAACGATTGGCCAAACTATCTGACGGAGTAGCAGTATTGAAGGTTGGCGGCACAAGTG

ATGTTGAAGTCAATGAGAAGAAGGACAGAGTTACCGATGCCCTGAACGCGACCCGTGCTGCTGTAGAGGA

AGGCATTGTTCCAGGCGGTGGGTGTGCATTGCTTCGCTGCATTCCAGCATTAGACGCTTTAAAACCAGCC

AACGAAGATCAGAAAATTGGCATTGAAATAATAAAGAGAACATTGAAAATTCCAGCAATGACTATTGCAA

AGAATGCAGGTGTTGAAGGGTCGTTAATAGTTGAAAAAATACTGCAGAGTTCATCAGAAGTTGGCTATGA

TGCGATGCTTGGGGAATTTGTAAATATGGTAGAAAAAGGAATCATAGACCCAACAAAGGTTGTGAGAACG

GCTCTGATGGACGCTGCAGGTGTTGCTTCTCTCTTATCAACGGCAGAAGCAGTAGTGACTGAAGTTCCTA

AAGAGGAAAAAGAGCCAGCAATGGGAGGAATGGGAGGGATGGGTGGAGGAATGGGAGGTGGCATGTTCTA

ATTCCTGGGATATTGATGCATCATGAACTGTGCTGTTTAAGACTGACAGTTCTGTCTTAAAAACTTCAGA

TGTCAGTGAGAGAGGATGGATAGTGACTGAAGTGAGGCTGGTGTTCAAAAGAATCACTGTAACCATCAGT

TACTGGATTTCATTTAACACACGTGTAATTGTTTACAGTCATTGTCCATGCCTACAGATAATTTATTTTG

TATTTTTTGAATAAAGACATTTGTACATTCCTGA

Protein Sequencing

>NP_001103255.2 heat shock protein HSP 90-alpha [Gallus gallus]

MPEAVQTQDQPMEEEVETFAFQAEIAQLMSLIINTFYSNKEIFLRELISNSSDALDKIRYESLTDPSKLD

SGKDLKINLIPNKHDRTLTIVDTGIGMTKADLVNNLGTIAKSGTKAFMEALQAGADISMIGQFGVGFYSA

YLVAEKVTVITKHNDDEQYAWESSAGGSFTVRLDNGEPLGRGTKVILHLKEDQTEYLEERRIKEIVKKHS

QFIGYPIRLFVEKERDKEVSDDEAEEKEEEKEEKEEKTEDKPEIEDVGSDEEEEKKDGDKKKKKKIKEKY

IDEEELNKTKPIWTRNPDDITNEEYGEFYKSLTNDWEDHLAVKHFSVEGQLEFRALLFVPRRAPFDLFEN

RKKKNNIKLYVRRVFIMDNCEELIPEYLNFMRGVVDSEDLPLNISREMLQQSKILKVIRKNLVKKCLELF

TELAEDKENYKKFYEQFSKNIKLGIHEDSQNRKKLSELLRYYTSASGDEMVSLKDYCTRMKENQKHVYYI

TGETKDQVANSAFVERLRKHGLEVIYMIEPIDEYCVQQLKEFEGKTLVSVTKEGLELPEDEEEKKKQEEK

KAKFENLCKIMKDILEKKVEKVVVSNRLVTSPCCIVTSTYGWTANMERIMKAQALRDNSTMGYMAAKKHL

EINPDHSIIETLRQKAEADKNDKSVKDLVILLYETALLSSGFSLEDPQTHANRIYRMIKLGLGIDEDDTA

AEEASPAVTEEMPPLEGDDDTSRMEEVD

>NP_001006686.1 heat shock 70 kDa protein [Gallus gallus]

MSGKGPAIGIDLGTTYSCVGVFQHGKVEIIANDQGNRTTPSYVAFTDTERLIGDAAKNQVAMNPTNTIFD

AKRLIGRKYDDPTVQSDMKHWPFRVVNEGGKPKVQVEYKGEMKTFFPEEISSMVLTKMKEIAEAYLGKKV

ETAVITVPAYFNDSQRQATKDAGTITGLNVMRIINEPTAAAIAYGLDKKGTRAGEKNVLIFDLGGGTFDV

SILTIEDGIFEVKSTAGDTHLGGEDFDNRMVNRFVEEFKGKHKRDNAGNKRAVRRLRTACERARRTLSSS

TQASIEIDSLFEGIDFYTSITRARFEELNADLFRGTLEPVEKALRDAKLDKGQIQEIVLVGGSTRIPKIQ

KLLQDFFNGKELNKSINPDEAVAYGAAVQAAILMGDKSENVQDLLLLDVTPLSLGIETAGGVMTALIKRN

TTIPTKQTQTFTTYSDNQSSVLVQVYEGERAMTKDNNLLGKFDLTGIPPAPRGVPQIEVTFDIDANGILN

VSAVDKSTGKENKITITNDKGRLSKDDIDRMVQEAEKYKAEDEANRDRVGAKNSLESYTYNMKQTVEDEK

LKGKISDQDKQKVLDKCQEVISSLDRNQMAEKEEYEHKQKELEKLCNPIVTKLYQGAGGAGAGGSGGPTI

EEVD

>NP_001012934.1 60 kDa heat shock protein, mitochondrial precursor [Gallus gallus]

MLRLPAVLRQIRPVSRALAPHLTRAYAKDVKFGADARALMLQGVDLLADAVAVTMGPKGRTVIIEQSWGS

PKVTKDGVTVAKAIDLKDKYKNIGAKLVQDVANNTNEEAGDGTTTATVLARAIAKEGFEKISKGANPVEI

RRGVMLAVDAITAELKKLSKPVTTPEEIAQVATISANGDQEIGNIISDAMKKVGRKGVITVKDGKTLNDE

LEIIEGMKFDRGYISPYFINTAKGQKCEFQDAYVLISEKKISSVQSIVPALEIANSHRKPLVIIAEDVDG

EALSTLVLNRLKVGLQVVAVKAPGFGDNRKNQLKDMAIATGGAVFGEEGLSLNVEDIQPHDFGKVGEVIV

TKDDTMLLKGKGEKAQIEKRIQEIIEQLEVTTSEYEKEKLNERLAKLSDGVAVLKVGGTSDVEVNEKKDR

VTDALNATRAAVEEGIVPGGGCALLRCIPALDALKPANEDQKIGIEIIKRTLKIPAMTIAKNAGVEGSLI

VEKILQSSSEVGYDAMLGEFVNMVEKGIIDPTKVVRTALMDAAGVASLLSTAEAVVTEVPKEEKEPAMGG

MGGMGGGMGGGMF
