# Supplementary material for: HSP expression depends on its molecular construction and different organs of the chicken: a meta-analysis
Source: Sci Rep. 2022 Sep 1;12:14901. doi: 10.1038/s41598-022-18985-0 (PMC9437049; doi:10.1038/s41598-022-18985-0)
Supplement: Supplementary file 3 — Supplementary Information 3. [file 41598_2022_18985_MOESM3_ESM.docx]

**Supplementary Information**


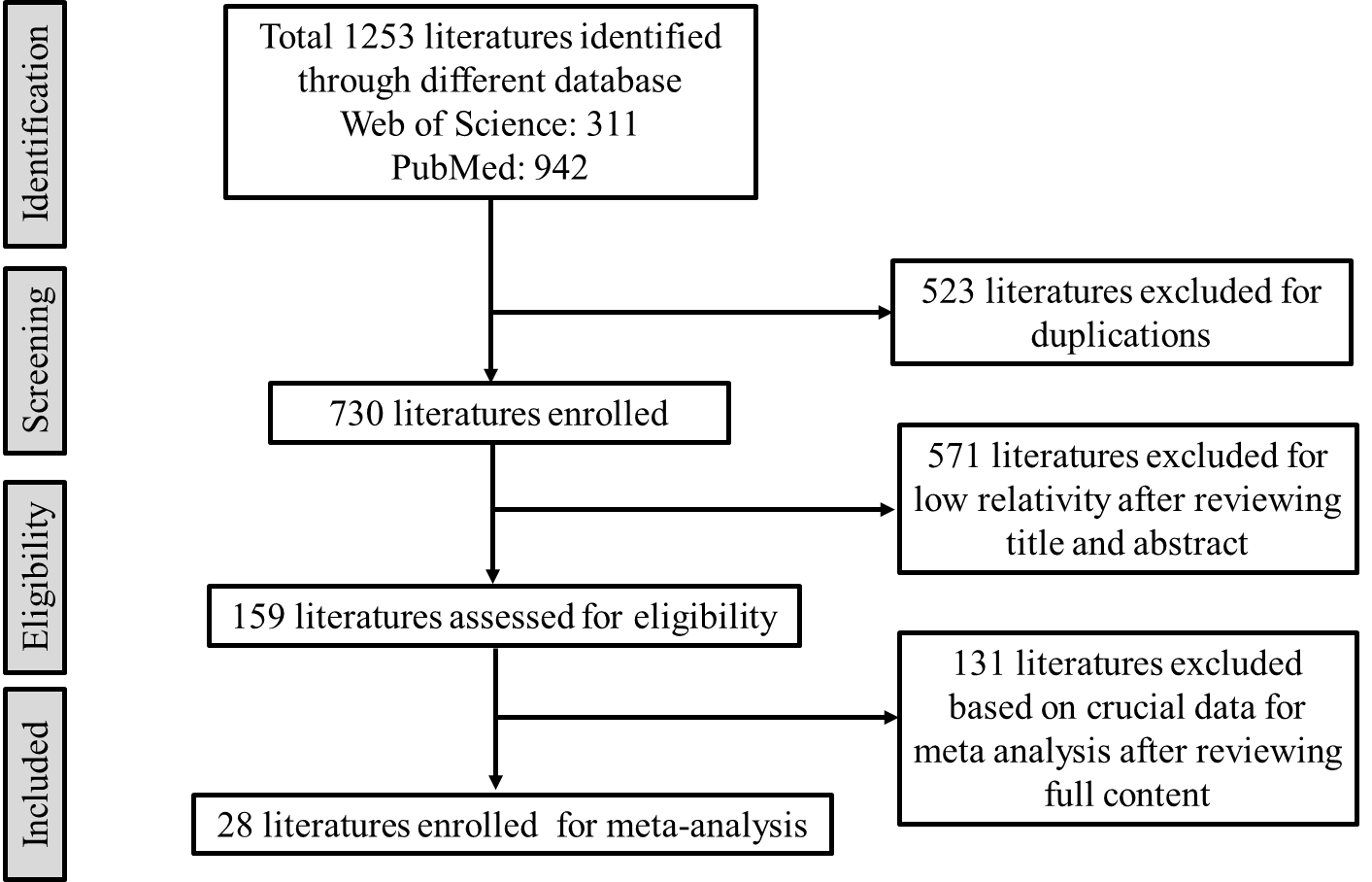


**Fig. S1** The study selection flowchart. Randomized controlled trials published before February 2022 in the PubMed, Goggle scholar, and Web of Science databases were searched following the Preferred Reporting Items for Systematic Review and Meta-Analysis (PRISMA) guidelines.


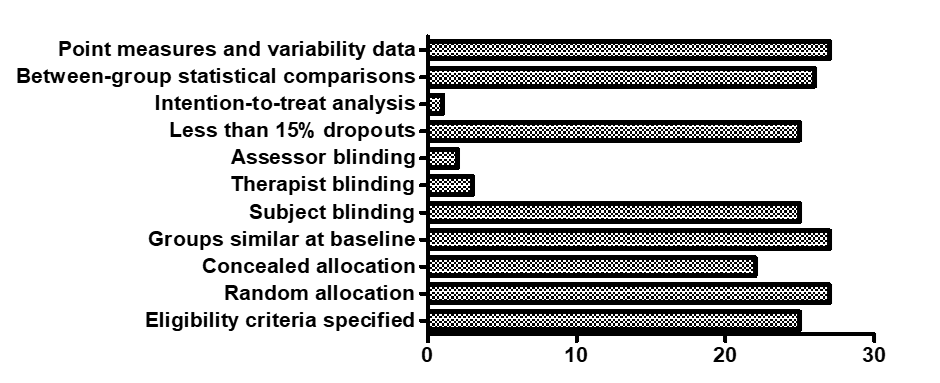


**Fig. S2** Number of studies meeting individual PEDro [Physiotherapy Evidence Database] criteria.


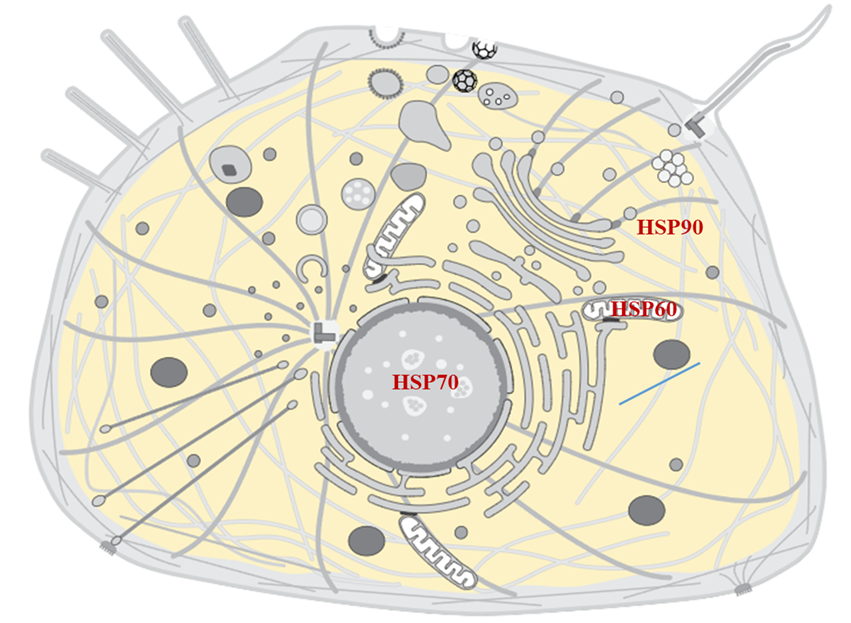


**Fig. S3** Subcellular location of Heat Shock Proteins. The figures were created by the biorender (https://biorender.com/).

**Table S1.** Characterization of selected published papers.

| Study | Strain | No. of broiler/ group | Age for experiment | Thermoneutral  temperature | Heat stress temperature |
| --- | --- | --- | --- | --- | --- |
| Uerlings et al., 2018 ^1^ | Arbor  Acres | 40 | 9 day | 30±1°C | 39±1°C |
| Al-Zghoul et al., 2020 ^2^ | Cobb | Not mention | Embryo | 37.8±1°C | 39±1°C |
| Varasteh et al., 2015 ^3^ | Ross | 10 | 15 day | 24±1°C | 38±1°C |
| Yang et al., 2021 ^4^ | Shan Ma ducks | 60 | 60 day | 24±2°C | 39±1°C |
| Siddiqui et al., 2020 ^5^ | Ross-308 | 120 | 21 day | 22±2°C | 34±1°C |
| Tabler et al., 2020 ^6^ | Broiler | 50 | 15 day | 24°C | 36°C |
| Yu et al., 2021 ^7^ | Wenchang  chicks | 50 | 14 day | 28.69 ± 0.18°C | 40 ± 0.50°C |
| Hasan Siddiqui et al., 2020 ^8^ | Ross-308 | 120 | 21day | 22±1°C | 34 ± 1°C |
| Albokhadaim et al., 2019 ^9^ | Ross 308 | 50 | 42 day | 22°C | 34°C |
| Al-Zghoul et al., 2015 ^10^ | Cobb | 20 | 24 day | 21±1°C | 43±1°C |
| Zhang et al., 2016 ^11^ | Ross-308 | 90 | 21 day | 25 ± 1°C | 40 ± 1°C |
| Tang et al., 2018 ^12^ | Not mention | 50 | 7 day | 25 ± 2°C | 42±1°C |
| Santos et al., 2019 ^13^ | Ross | 80 | 21 day | 21±1°C | 37±2°C |
| Yang et al., 2016 ^14^ | Julia | 15 | 12 day | 30 ±1◦C | 40 ± 1◦C |
| Han et al., 2019 ^15^ | Chunky | 18 | 9 day | 28 ± 1°C | 35 ± 1◦C |
| Rajaei-Sharifabadi et al., 2017 ^16^ | Cobb 500 | 36 | 35 day | 25 °C | 35 °C |
| Rajaei-Sharifabadi et al., 2017 ^17^ | Cobb 500 | 20 | 22 day | 25 °C by | 35 °C |
| Greene et al., 2021 ^18^ | Cobb 500 | Not mention | 24 day | 24 °C | 35 °C |
| Vinoth et al., 2015 ^19^ | Not mention | 15 | Not mention | 25 ±1°C | 35 ±1°C |
| Miao et al., 2021 ^20^ | Arbor Acres | 36 | 27 | 26 °C | 35 °C |
| Madkour et al., 2021 ^21^ | Cobb 500 | 60 | 22 | 25 ± 1°C | 39 °C |
| Al Wakeel et al., 2019 ^22^ | Ross 308 | 13 | 21 | 22 ±2° C | 33 ±1 C° |
| Kang and Shim, 2021 ^23^ | Ross | 48 | 28 | 24 °C | 40 °C |
| Roushdy et al.,2018 ^24^ | Cobb | 50 | 22 | 24 ± 2 °C | 36 °C |
| Belal et al., 2018 ^25^ | Ross | 20 | 21 | 24°C | 34 °C |
| Al-Zghoul, 2018 ^26^ | Ross 315 | Not mention | 14 | 25 ± 1 °C | 41 °C |
| Nguyen et al., 2017 ^27^ | Quails | Not mention | 28 | 24°C | 37 °C |

**References**

1 Uerlings, J. *et al.* Heat exposure affects jejunal tight junction remodeling independently of adenosine monophosphate-activated protein kinase in 9-day-old broiler chicks. *Poult Sci* **97**, 3681-3690, doi:10.3382/ps/pey229 (2018).

2 Al-Zghoul, M. B. & Mohammad Saleh, K. M. Effects of thermal manipulation of eggs on the response of jejunal mucosae to posthatch chronic heat stress in broiler chickens. *Poult Sci* **99**, 2727-2735, doi:10.1016/j.psj.2019.12.038 (2020).

3 Varasteh, S., Braber, S., Akbari, P., Garssen, J. & Fink-Gremmels, J. Differences in Susceptibility to Heat Stress along the Chicken Intestine and the Protective Effects of Galacto-Oligosaccharides. *PLOS ONE* **10**, e0138975, doi:10.1371/journal.pone.0138975 (2015).

4 Yang, C. *et al.* Resveratrol sustains intestinal barrier integrity, improves antioxidant capacity, and alleviates inflammation in the jejunum of ducks exposed to acute heat stress. *Poult Sci* **100**, 101459, doi:10.1016/j.psj.2021.101459 (2021).

5 Siddiqui, S. H., Kang, D., Park, J., Khan, M. & Shim, K. Chronic heat stress regulates the relation between heat shock protein and immunity in broiler small intestine. *Scientific Reports* **10**, 18872, doi:10.1038/s41598-020-75885-x (2020).

6 Tabler, T. W. *et al.* Intestinal Barrier Integrity in Heat-Stressed Modern Broilers and Their Ancestor Wild Jungle Fowl. *Front Vet Sci* **7**, 249, doi:10.3389/fvets.2020.00249 (2020).

7 Yu, Z., Tian, J., Wen, J. & Chen, Z. Effects of Heat Stress on Expression of Heat Shock Proteins in the Small Intestine of Wenchang Chicks. *Brazilian Journal of Poultry Science* **23** (2021).

8 Hasan Siddiqui, S., Kang, D., Park, J., Choi, H. W. & Shim, K. Acute Heat Stress Induces the Differential Expression of Heat Shock Proteins in Different Sections of the Small Intestine of Chickens Based on Exposure Duration. *Animals* **10**, doi:10.3390/ani10071234 (2020).

9 Albokhadaim, I. F., Althnaian, T. A. & El-Bahr, S. M. Gene expression of heat shoc kproteins/factors (HSP60, HSP70, HSP90, HSF-1, HSF-3) and antioxidant enzyme activities in heat stressed broilers treated with vitamin C. *Pol J Vet Sci* **22**, 565-572, doi:10.24425/pjvs.2019.129965 (2019).

10 Al-Zghoul, M. B. *et al.* Thermal manipulation during broiler chicken embryogenesis: Effect on mRNA expressions of Hsp108, Hsp70, Hsp47 and Hsf-3 during subsequent post-hatch thermal challenge. *Res Vet Sci* **103**, 211-217, doi:10.1016/j.rvsc.2015.10.015 (2015).

11 Zhang, X. H. *et al.* The association of Hsp90 expression induced by aspirin with anti-stress damage in chicken myocardial cells. *J Vet Sci* **17**, 35-44, doi:10.4142/jvs.2016.17.1.35 (2016).

12 Tang, S., Yin, B., Xu, J. & Bao, E. Rosemary Reduces Heat Stress by Inducing CRYAB and HSP70 Expression in Broiler Chickens. *Oxid Med Cell Longev* **2018**, 7014126, doi:10.1155/2018/7014126 (2018).

13 Santos, R. R. *et al.* Effects of a feed additive blend on broilers challenged with heat stress. *Avian Pathol* **48**, 582-601, doi:10.1080/03079457.2019.1648750 (2019).

14 Yang, H. *et al.* Chronic oral administration of pine bark extract (flavangenol) attenuates brain and liver mRNA expressions of HSPs in heat-exposed chicks. *J Therm Biol* **60**, 140-148, doi:10.1016/j.jtherbio.2016.06.014 (2016).

15 Han, G. *et al.* Effects of in ovo feeding of L-leucine on amino acids metabolism and heat-shock protein-70, and -90 mRNA expression in heat-exposed chicks. *Poult Sci* **98**, 1243-1253, doi:10.3382/ps/pey444 (2019).

16 Rajaei-Sharifabadi, H. *et al.* Noni (Morinda citrifolia) Modulates the Hypothalamic Expression of Stress- and Metabolic-Related Genes in Broilers Exposed to Acute Heat Stress. *Front Genet* **8**, 192, doi:10.3389/fgene.2017.00192 (2017).

17 Rajaei-Sharifabadi, H. *et al.* Surface wetting strategy prevents acute heat exposure-induced alterations of hypothalamic stress- and metabolic-related genes in broiler chickens. *J Anim Sci* **95**, 1132-1143, doi:10.2527/jas.2016.1290 (2017).

18 Greene, E. S. *et al.* Protective effects of the phytogenic feed additive “comfort” on growth performance via modulation of hypothalamic feeding- and drinking-related neuropeptides in cyclic heat-stressed broilers. *Domestic Animal Endocrinology* **74**, 106487, doi:<https://doi.org/10.1016/j.domaniend.2020.106487> (2021).

19 Vinoth, A., Thirunalasundari, T., Tharian, J. A., Shanmugam, M. & Rajkumar, U. Effect of thermal manipulation during embryogenesis on liver heat shock protein expression in chronic heat stressed colored broiler chickens. *Journal of Thermal Biology* **53**, 162-171, doi:<https://doi.org/10.1016/j.jtherbio.2015.10.010> (2015).

20 Miao, Q. X. *et al.* Acute heat stress alters the expression of genes and proteins associated with the unfolded protein response pathway in the liver of broilers. *Br Poult Sci*, 1-8, doi:10.1080/00071668.2021.1969644 (2021).

21 Madkour, M. *et al.* Hepatic expression responses of DNA methyltransferases, heat shock proteins, antioxidant enzymes, and NADPH 4 to early life thermal conditioning in broiler chickens. *Italian Journal of Animal Science* **20**, 433-446, doi:10.1080/1828051X.2021.1890645 (2021).

22 Al Wakeel, R. A., Saad, M. F., Abdel Azeez, A., Elkhiat, F. & Shukry, M. Both experimental hypo- and hyper-thyroidism exacerbate the adverse effects of chronic heat stress in broilers. *Br Poult Sci* **60**, 330-339, doi:10.1080/00071668.2019.1602248 (2019).

23 Kang, D. & Shim, K. Early heat exposure effect on the heat shock proteins in broilers under acute heat stress. *Poult Sci* **100**, 100964, doi:10.1016/j.psj.2020.12.061 (2021).

24 Roushdy, E. M., Zaglool, A. W. & El-Tarabany, M. S. Effects of chronic thermal stress on growth performance, carcass traits, antioxidant indices and the expression of HSP70, growth hormone and superoxide dismutase genes in two broiler strains. *J Therm Biol* **74**, 337-343, doi:10.1016/j.jtherbio.2018.04.009 (2018).

25 Belal, S., Kang, D., Cho, E., Park, G. & Shim, K. Taurine reduces heat stress by regulating the expression of heat shock proteins in broilers exposed to chronic heat. *brazilian journal of poultry science* **20**, 479-486 (2018).

26 Al-Zghoul, M. B. Thermal manipulation during broiler chicken embryogenesis increases basal mRNA levels and alters production dynamics of heat shock proteins 70 and 60 and heat shock factors 3 and 4 during thermal stress. *Poult Sci* **97**, 3661-3670, doi:10.3382/ps/pey225 (2018).

27 Nguyen, P. H. *et al.* Acute Heat Stress Alters the Expression of Orexin System in Quail Muscle. *Front Physiol* **8**, 1079, doi:10.3389/fphys.2017.01079 (2017).
